# Supplementary material for: Rapid P-TEFb-dependent transcriptional reorganization underpins the glioma adaptive response to radiotherapy
Source: Nat Commun. 2024 May 30;15:4616. doi: 10.1038/s41467-024-48214-3 (PMC11139976; doi:10.1038/s41467-024-48214-3)
Supplement: Supplementary file 1 — Supplementary Information [file 41467_2024_48214_MOESM1_ESM.docx]

**Supplementary Data**

**
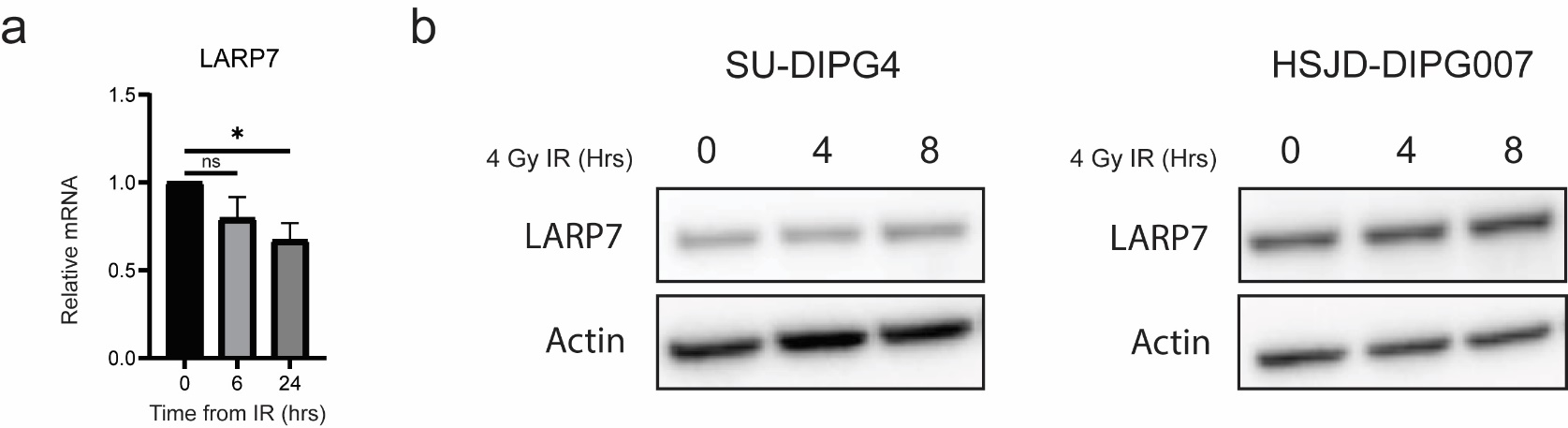
**

**Supplementary Figure 1.** Immunoblot of LARP7 following IR exposure in SU-DIPG4 and HSJD-DIPG007. Data represent single experiment. Source data are provided as a Source Data file.

**
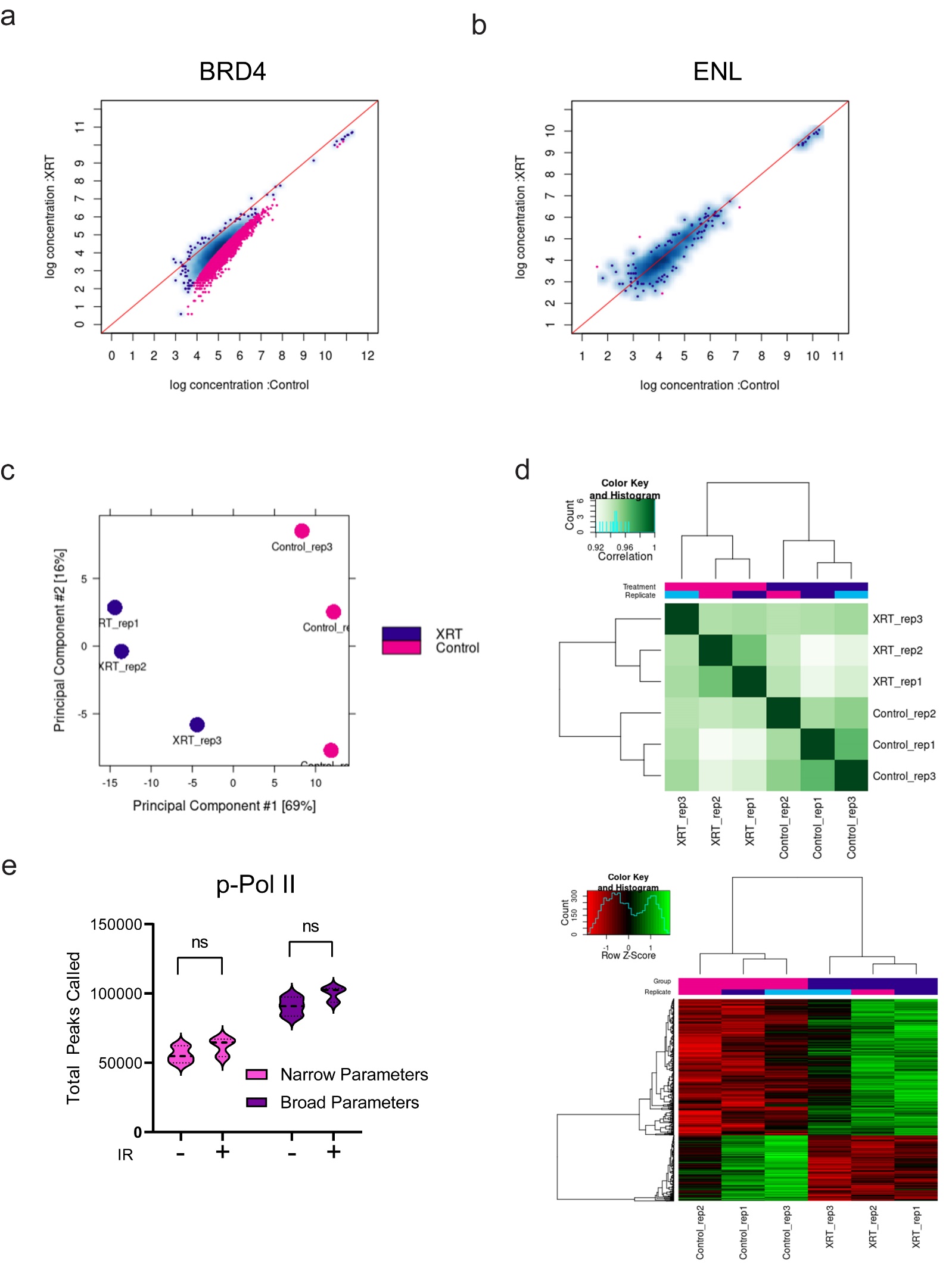
**

**Supplementary Figure 2.** P-TEFb and p-Pol II CUT&RUN reveals distinct occupancy changes following IR-exposure. **a.** Scatterplot of BRD4 CUT&RUN peaks compared between IR-exposed cells and untreated controls (n=2). Differentially bound peaks are indicated in pink. **b.** Scatterplot of ENL CUT&RUN peaks compared between IR-exposed cells and untreated controls (n=2). Differentially bound peaks are indicated in pink. **c.** Principle component analysis of differentially bound, reproducible p-Pol II peaks. **d.** Correlation heatmaps using occupancy caller score (top) and binding affinity (bottom) of differentially bound (pval <0.05) p-Pol II peak calls demonstrates concordance within sample replicates. **e.** Total peaks called using MACS2 narrow or broad peak parameters. Quantitative comparisons reflect two-tailed Student’s t-test.

**
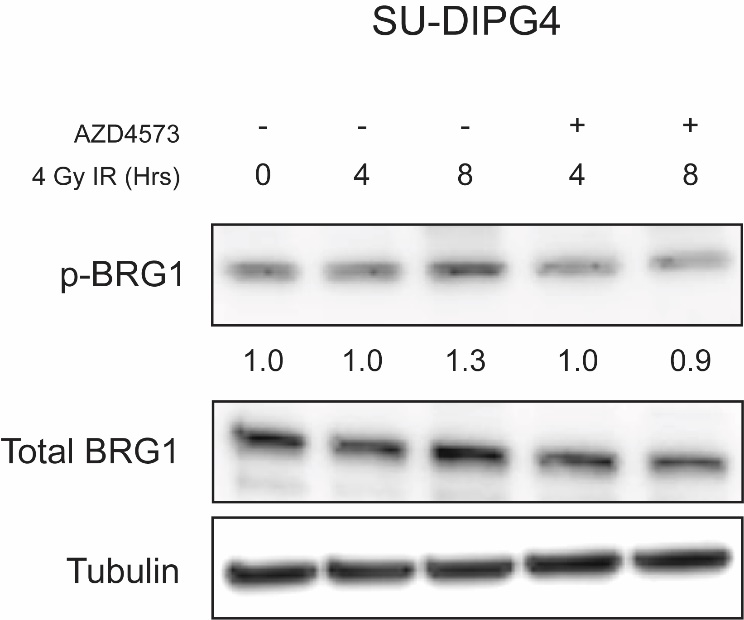
**

**Supplementary Figure 3.** Immunoblot of p-BRG1 (Ser1627/1631) following IR exposure in the presence or absence of AZD4573. Normalized ratio of quantified p-BRG1 to total BRG1 shown below. Data represent single experiment. Source data are provided as a Source Data file.

**
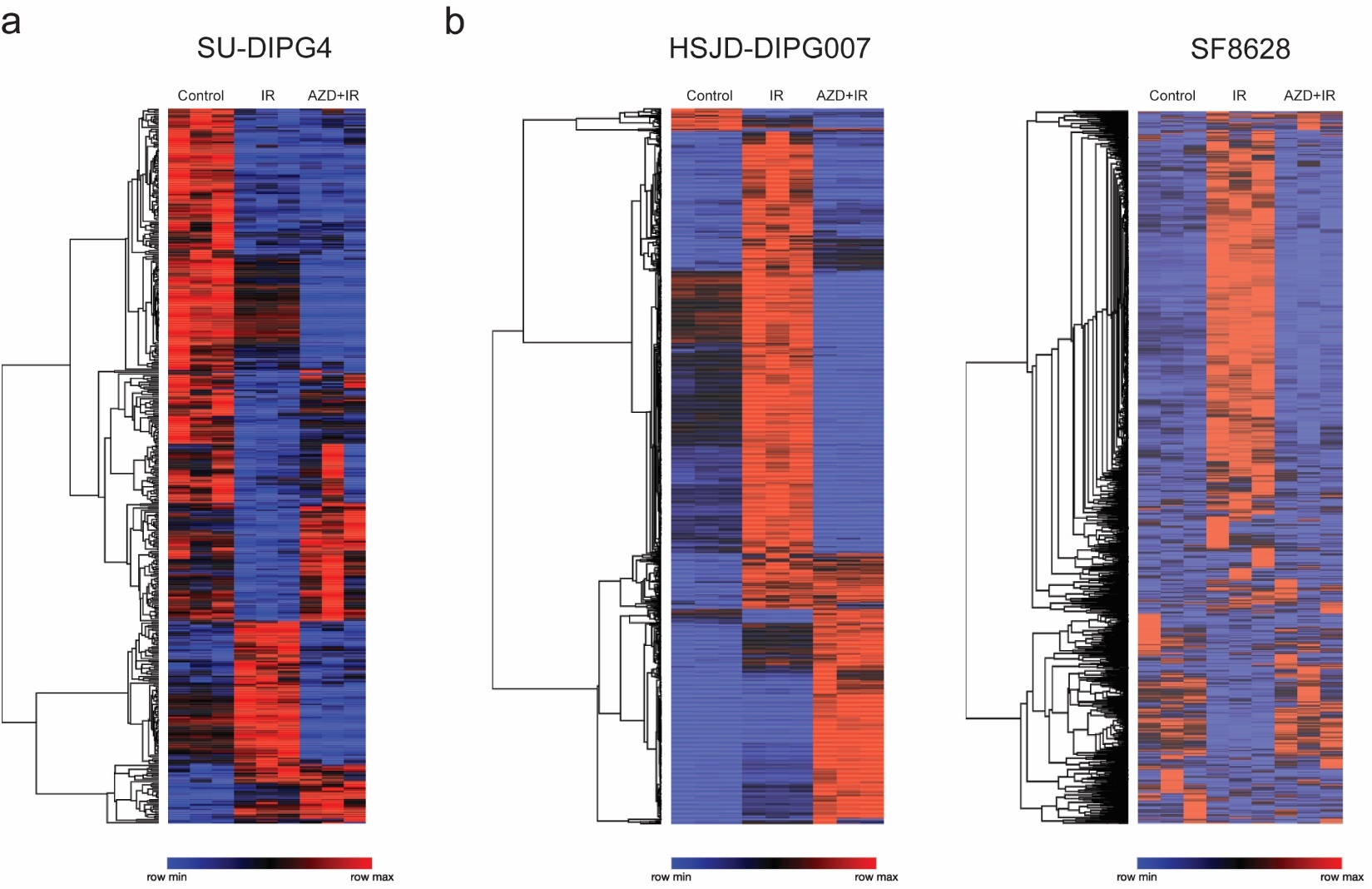
**

**Supplementary Figure 4.** IR-induced expression profiles of DMG cell lines. **a.** Heatmap of SU-DIPG4 gene expression LFC ≥ +/- 1.2 in control vs IR samples (n=3, padj <0.05). **b.** Unsupervised hierarchical clustering of HSJD-DIPG7 and SF8628 gene expression LFC ≥ +/- 1.2 in control vs IR samples (n=3, padj <0.05).


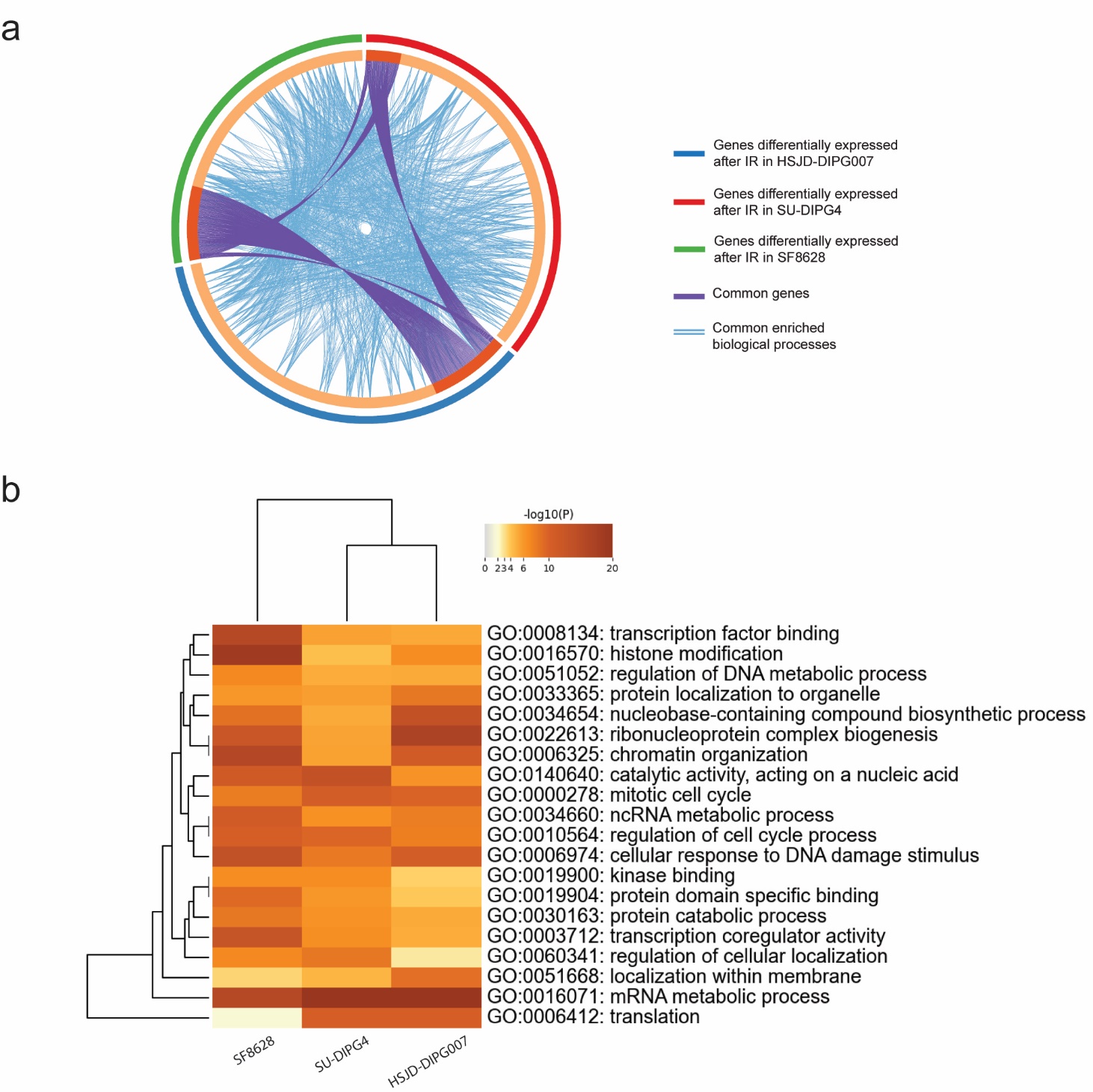


**Supplementary Figure 5.** IR-induced expression changes in pHGG models. **a.** Circos plot of genes upregulated in respective models following single IR exposure. Identical genes are indicated in purple, while common biological processes are indicated in blue. **b.** Unsupervised hierarchical clustering of common ontology terms from (a).


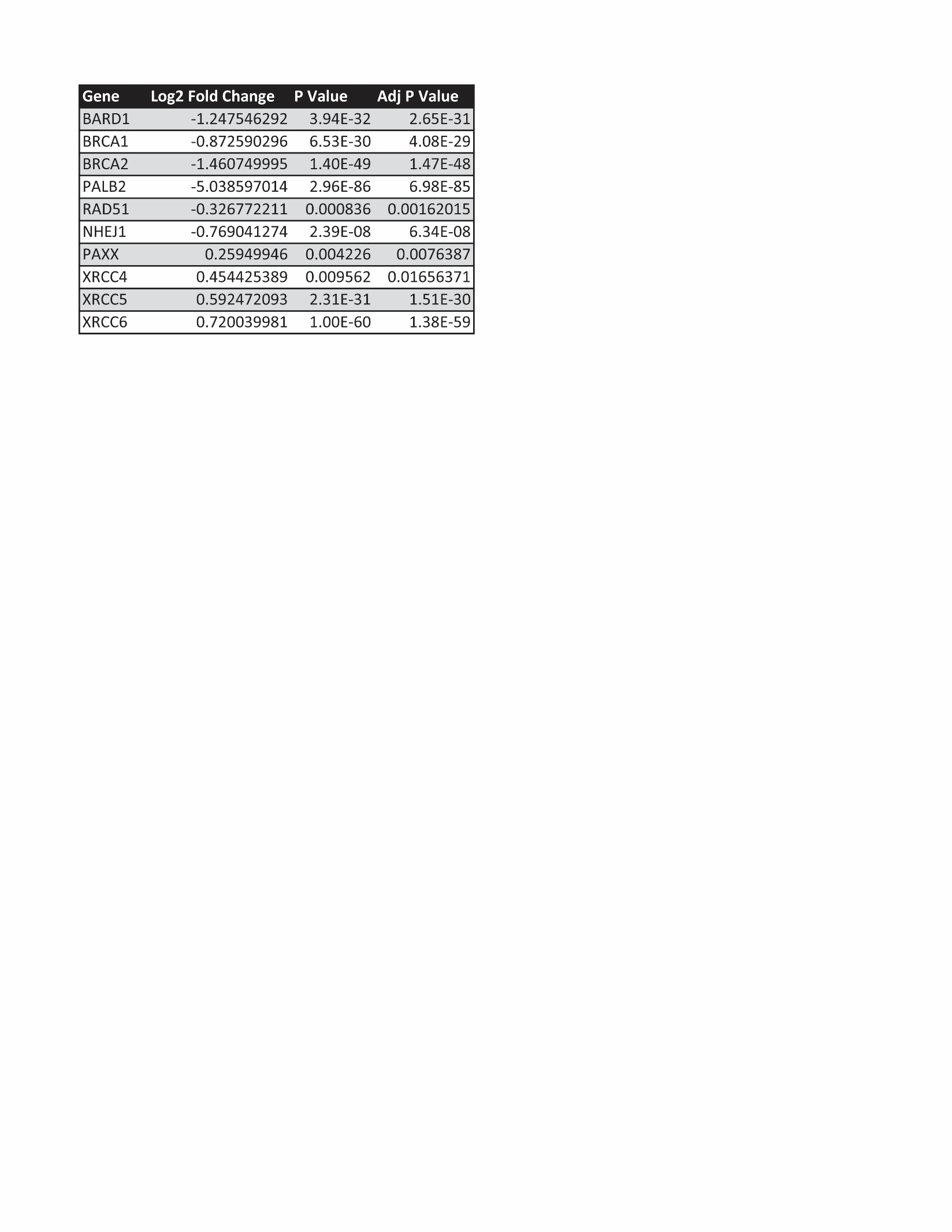


**Supplementary Table 1.** Log2 fold change of indicated genes from SU-DIPG4 RNA-seq, IR vs AZD+IR.


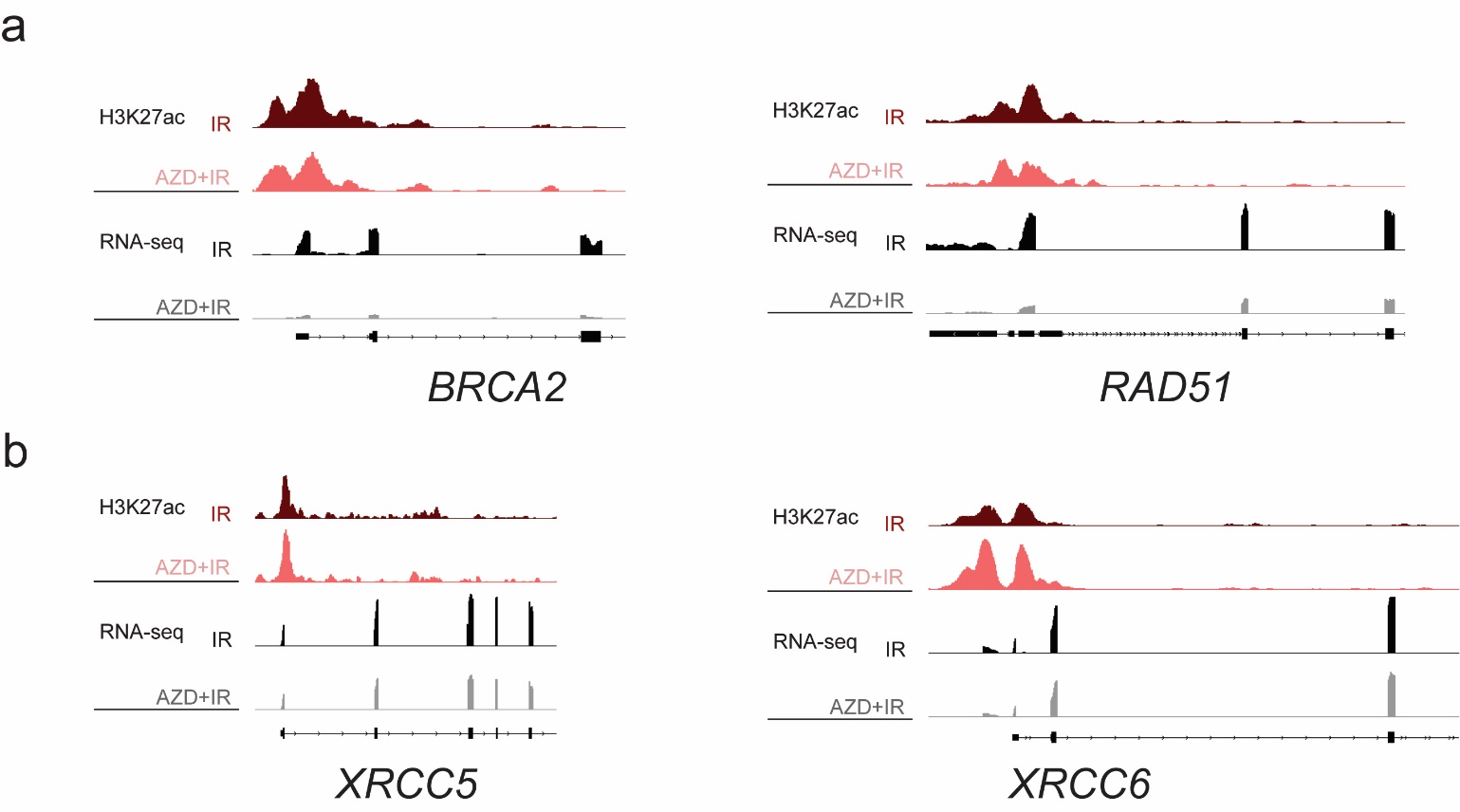


**Supplementary Figure 6.** Canonical HR and NHEJ gene expression changes following addition of AZD4573 to IR. **a.** Illustrative loci at HR genes *BRCA2* and *RAD51* promoters demonstrate modest decrease in H3K27ac occupancy with significant decrease in RNA-seq reads. Paired tracks reflect same data scale. **b.** Illustrative loci at NHEJ genes *XRCC5* and *XRCC6* promoters demonstrate stable H3K27ac occupancy and RNA-seq reads. Paired tracks reflect same data scale.


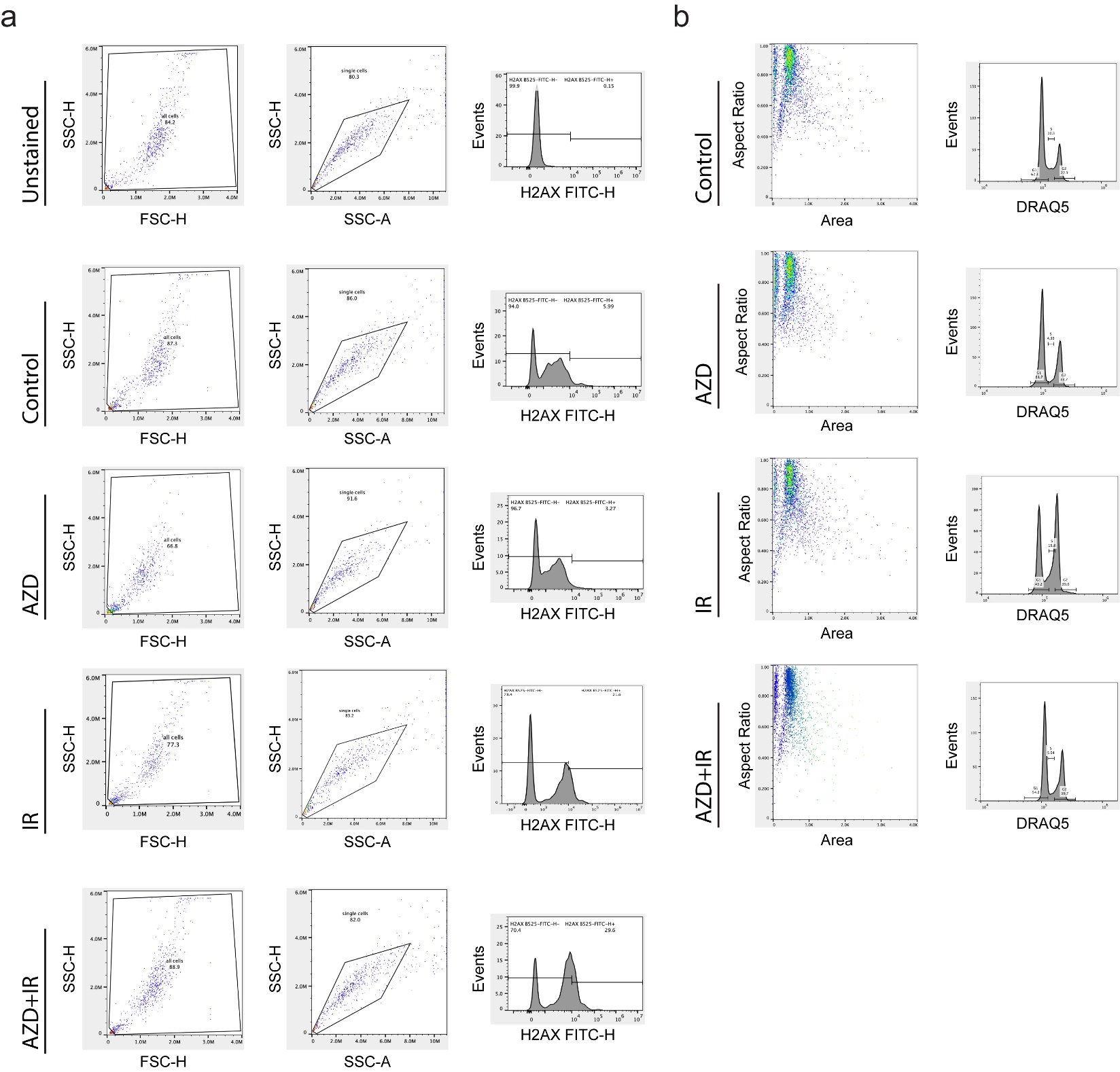


**Supplementary Figure 7.** Flow cytometry gating strategy. **a.** Gating strategy for yH2AX detection corresponding to Figure 4c-d. All viable cells are gated on FSC versus SSC (left) followed by gate for single cells on SSC-A versus SSC-H (center). Distribution of yH2AX intensity shown on right, with same cutoff used for all conditions. Images representative of n=3 biologically independent replicates per condition. **b.** Gating strategy for cell cycle analysis corresponding to Figure 4e-f. All events are captured without gating (left), with distribution of DRAQ5 intensity shown on right. Images representative of n=3 biologically independent replicates per condition.


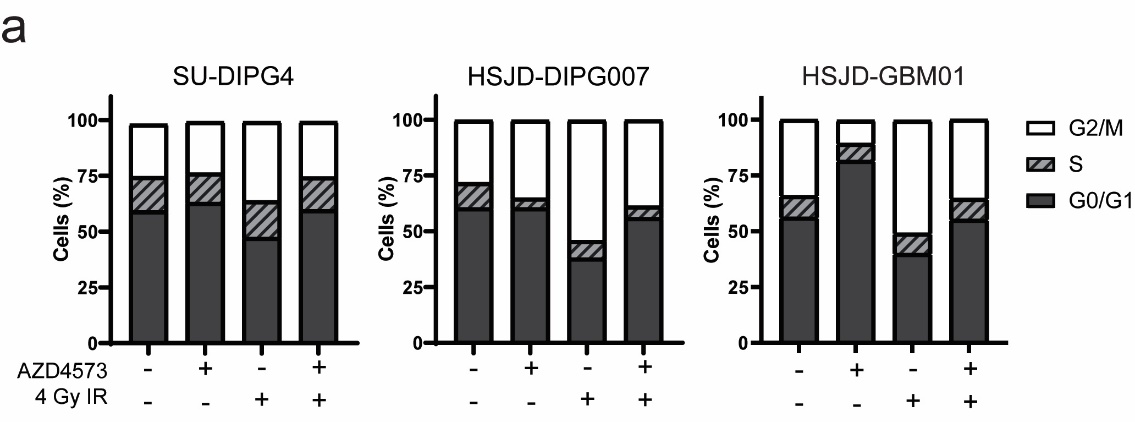


**Supplementary Figure 8.** Unsynchronized cell cycle analysis of AZD4573 and IR combinatorial therapy. **a.** Cell cycle distribution of indicated cultures after treatment with 8 nM AZD4573, IR, or combination. n=3 biologically independent replicates per cell line. Source data are provided as a Source Data file.


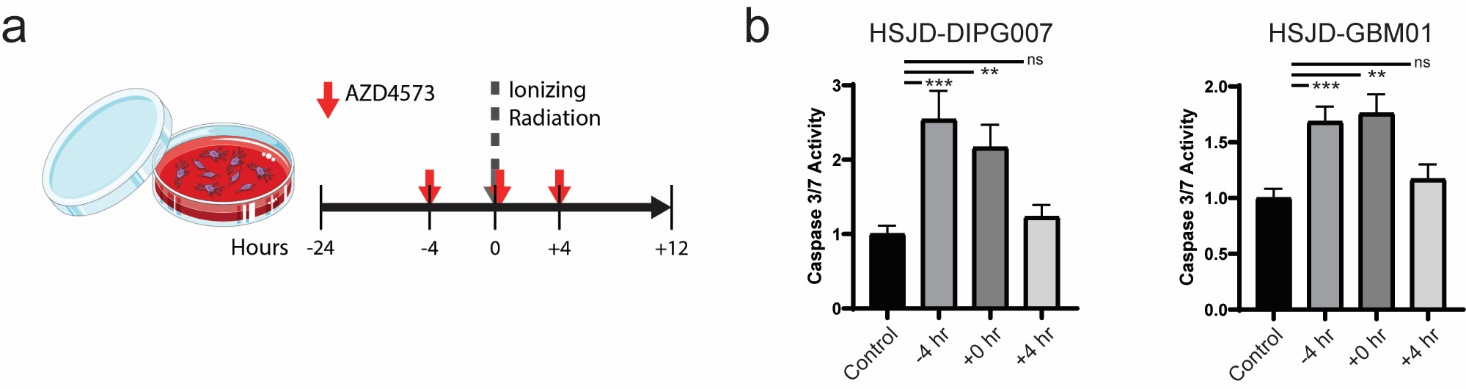


**Supplementary Figure 9.** Sequential optimization of AZD4573 and IR combinatorial therapy. **a.** Schematic of the experiment in which a fixed 6 nM dose of AZD4573 was added at various timepoints relative to single 4 Gy IR treatment. Caspase 3/7 activity was then measured at 12 hours following IR. **b.** Relative caspase 3/7 activity of HGG cultures treated with IR (control) and AZD4573 at indicated timepoints. Quantitative comparisons reflect two-tailed Student’s t-test (** p=<0.01, *** p=<0.001), mean ±SEM of n=9 biologically independent replicates. Source data are provided as a Source Data file.


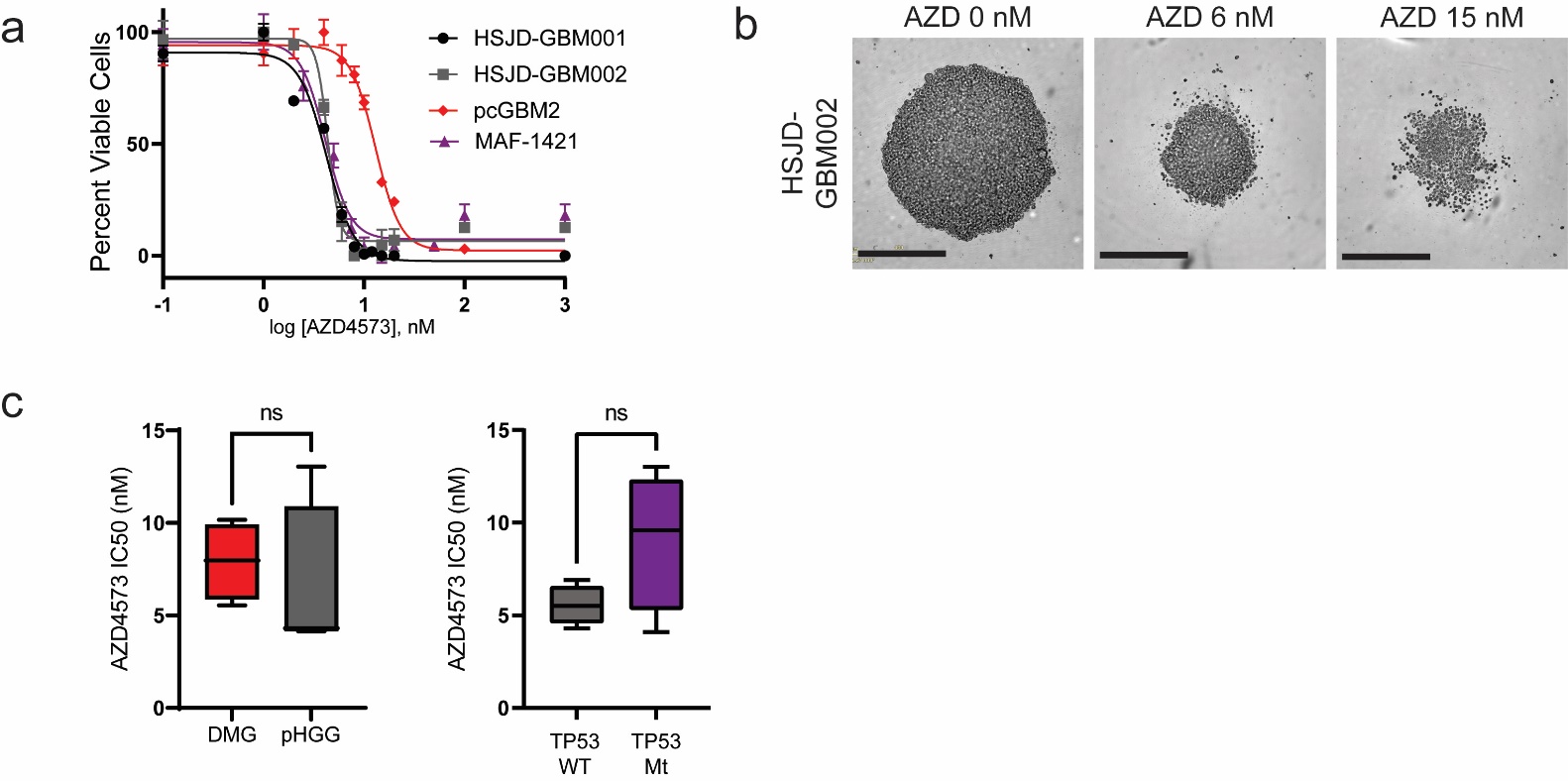


**Supplementary Figure 10.** AZD4573 demonstrates *in vitro* efficacy across the mutational spectrum of pHGG. **a.** Dose response curve (mean ±SEM) of AZD4573 across a panel of H3 WT (HSJD-GBM001 n=5, pcGBM2 n=6, MAF-1421 n=6) and H3G34R mutant (HSJD-GBM002 n=6) glioma cultures. Corresponding dose response curves in panel of H3K27M cultures previously published in (Dahl et al., 2020). **b.** Representative live cell imaging of HSJD-GBM002 neurosphere cultures at indicated concentrations of AZD4573 (scale bars, 400 µM)­­­. **c.** Mean half-maximal inhibitory concentration of AZD4573 in H3K27M-mutant cultures (DMG) in comparison to H3 WT or H3G34R-mutant (pHGG) (left, n=4 cell lines each) or *TP53* wild-type cultures versus *TP53* mutant (right, n=4 cell lines each). Quantitative comparison reflects two-tailed Student’s t-test. Box plots display interquartile range, median, and whisker (minimum to maximum). Source data are provided as a Source Data file.


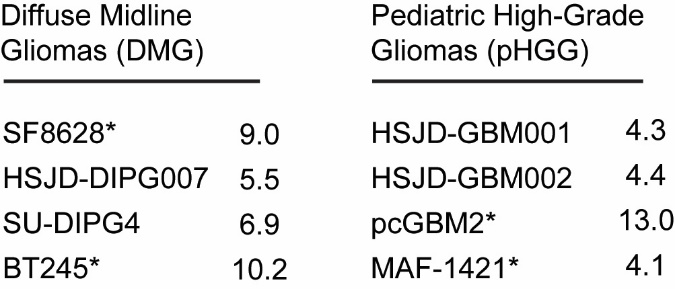


**Supplementary Table 2.** Individual culture models and half-maximal inhibitory concentration of AZD4573 in nM, * indicates *TP53* mutant status.


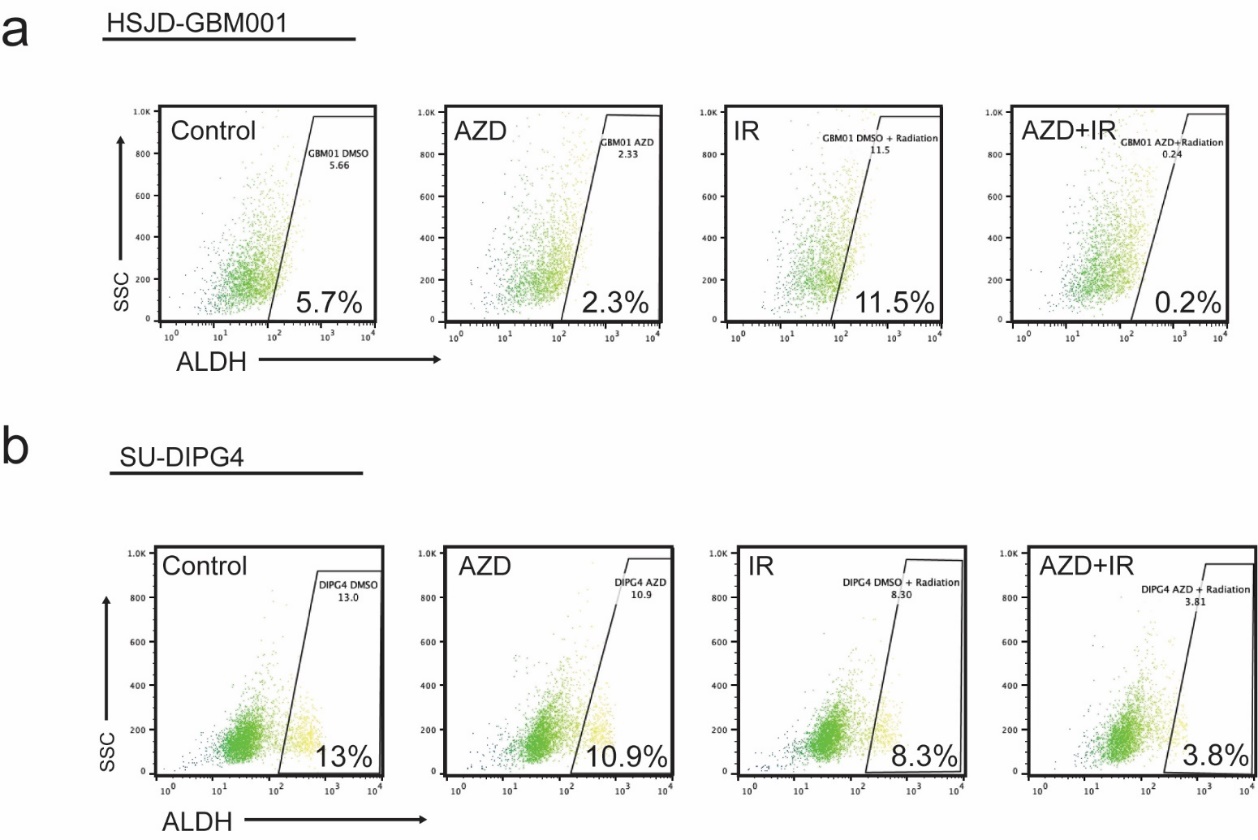


**Supplementary Figure 11.** AZD4573 and IR combinatorial therapy effectively deplete brain tumor initiating cell fraction within culture models. Brain tumor initiating cell fraction after DMSO control, AZD4573 (5nM), IR (4 Gy), or combination treatment as identified by ALDH expression in (**a**) H3 WT pHGG (HSJD-GBM001) and (**b**) H3K27M-mutant DMG (SU-DIPG4). n=2 biologically independent replicates per cell line.


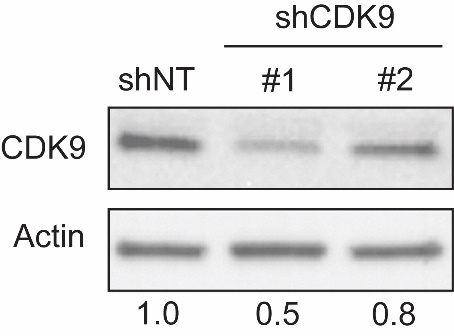


**Supplementary Figure 12.** CDK9-targeted shRNA knockdown efficacy. Western blot analysis for CDK9 following shRNA transduction, relative densitometry quantification shown below. Data represent single experiment. Source data are provided as a Source Data file.

­


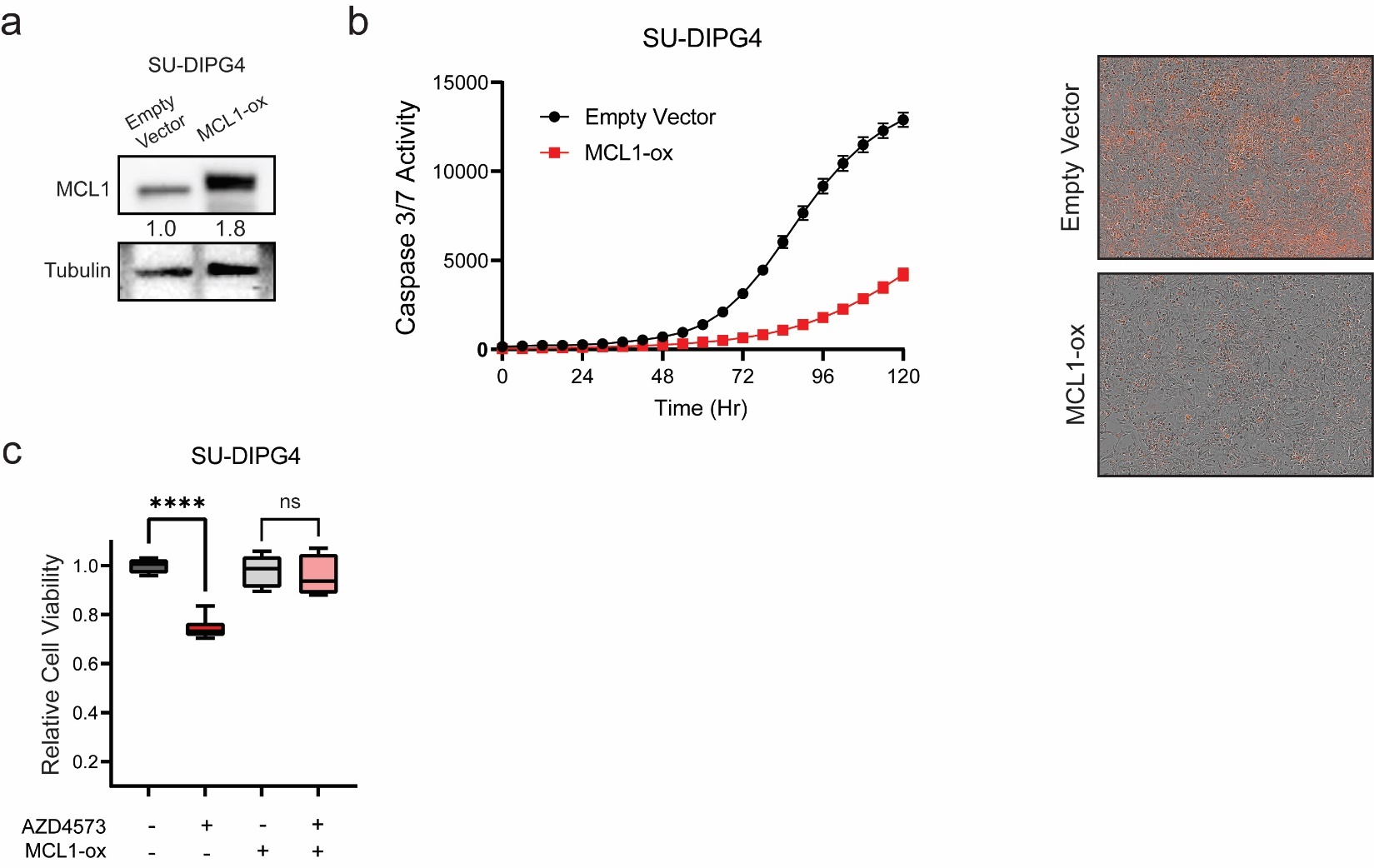


**Supplementary Figure 13.** Induction of apoptosis by AZD4573 is dependent on MCL1. **a.** Immunoblot for MCL1 in SU-DIPG4 transduced with MCL1 overexpression vector (MCL1-ox) or empty vector control. Data represent single experiment. **b.** Caspase 3/7 activity over time (left) following fixed 4nM dose of AZD4573. Error bars indicate SEM from minimum 3 biological replicates. Representative live-cell images from 96-hour timepoint shown on right. **c.** Cell viability following AZD4573 treatment with or without MCL1-ox. Quantitative comparison reflects two-tailed Student’s t-test (**** p=<0.0001). Box plots display interquartile range, median, and whisker (minimum to maximum). Source data are provided as a Source Data file.


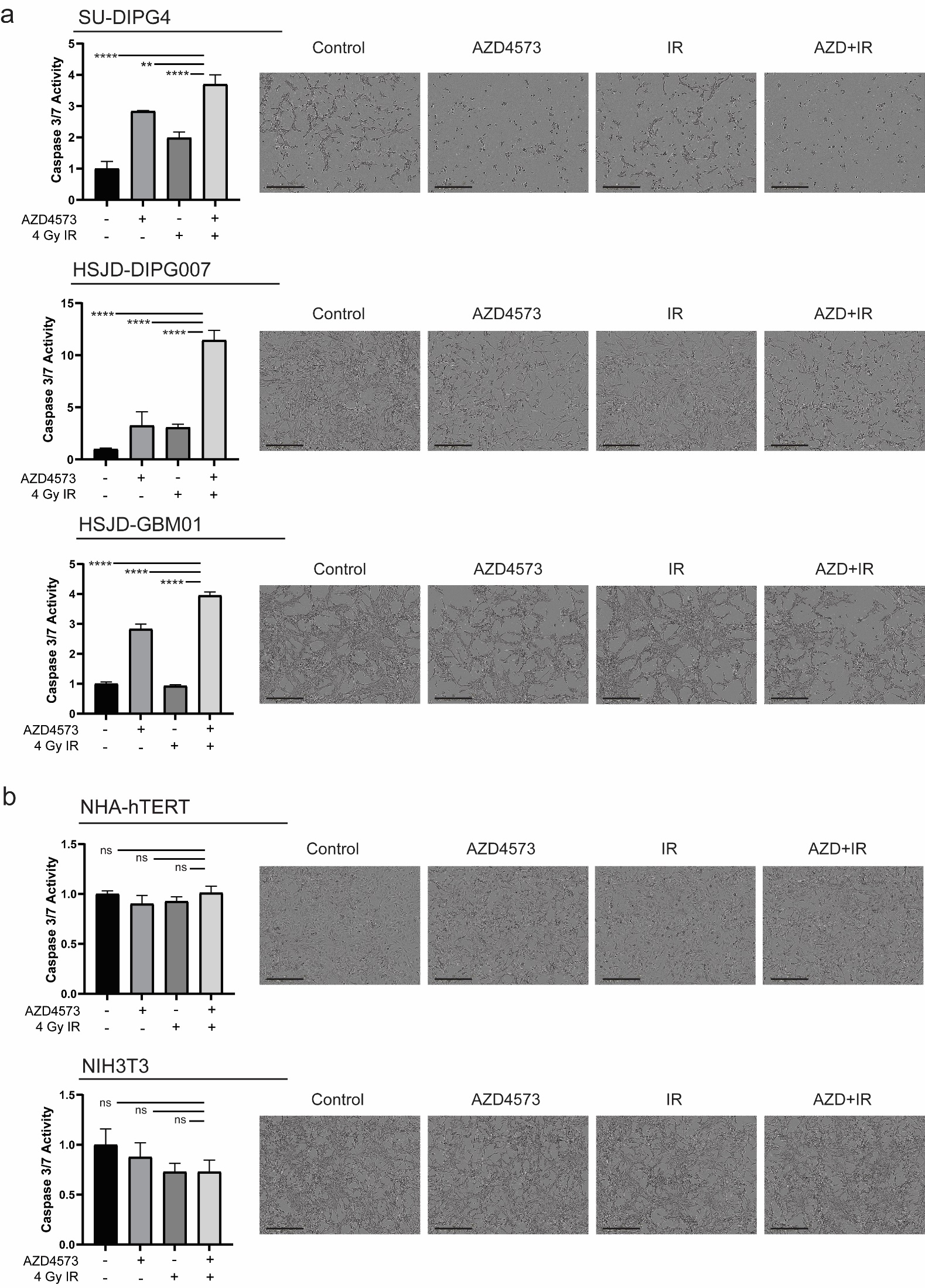


**Supplementary Figure 14.** Therapeutic index for CDK9i and IR in HGG cultures relative to normal controls. Caspase 3/7 activity (24 hours, left) and representative live-cell imaging (36 hours, right) of pediatric HGG cultures (**a**) or normal cell controls (**b**) after treatment with DMSO control, AZD4573, IR, or combination. Quantitative comparisons reflect two-tailed Student’s t-test (** p=<0.01, *** p=<0.001, **** p=<0.0001), mean ±SEM of n=4 biologically independent replicates. Source data are provided as a Source Data file.


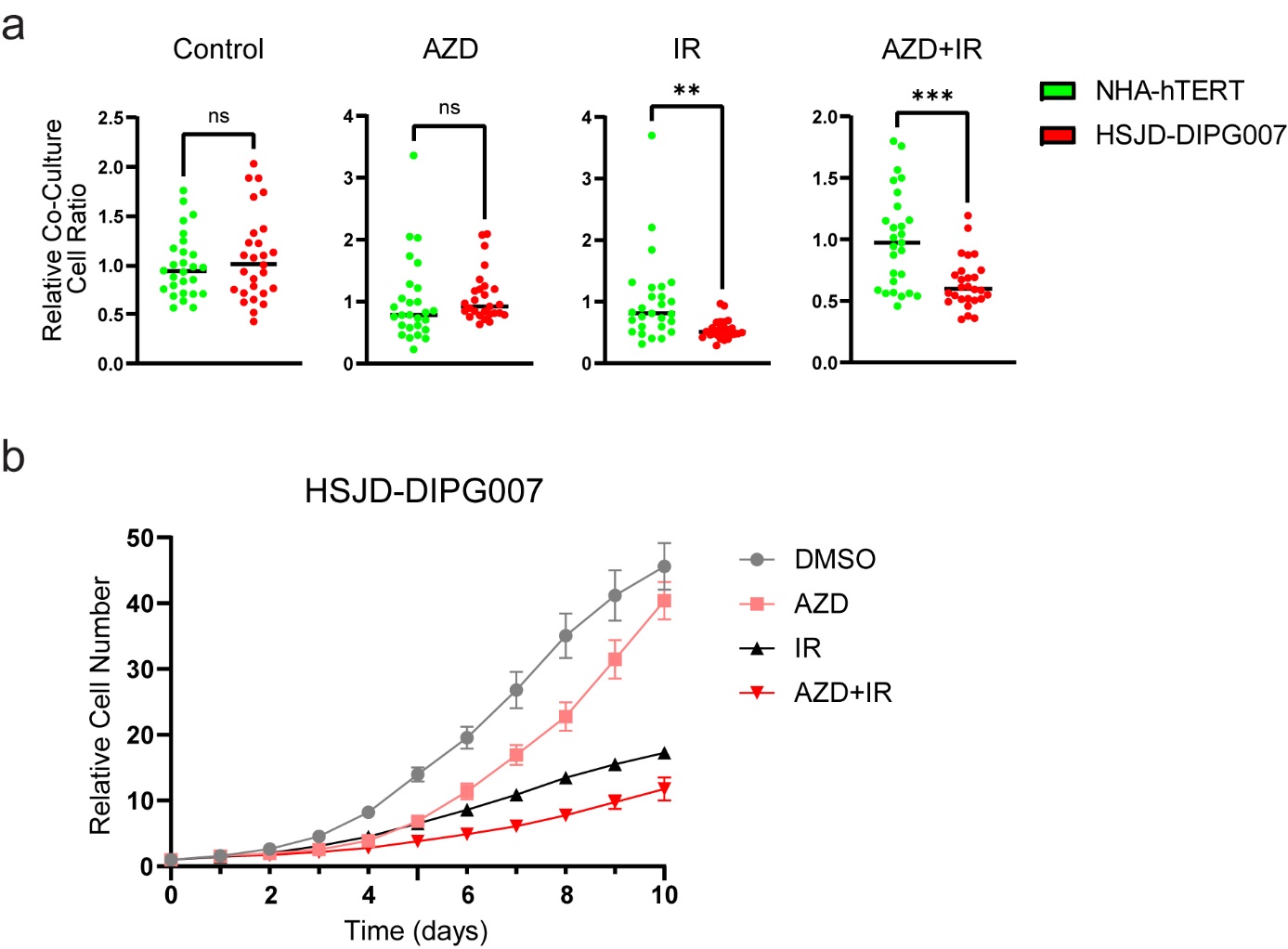


**Supplementary Figure 15.** Therapeutic index of combinatorial therapy within a co-culture system. **a.** Relative ratio quantification of co-cultured DIPG cell and astrocytes at day 10 following indicated treatments. Quantitative comparisons reflect two-tailed Student’s t-test (** p=<0.01, *** p=<0.001), n=6 biologically independent replicates imaged 4 fields per replicate. **b.** Relative number of HSJD-DIPG7 cells from co-culture system at indicated timepoints, shown as mean ± SEM of n=6 biologically independent replicates imaged 4 fields per replicate. Source data are provided as a Source Data file.


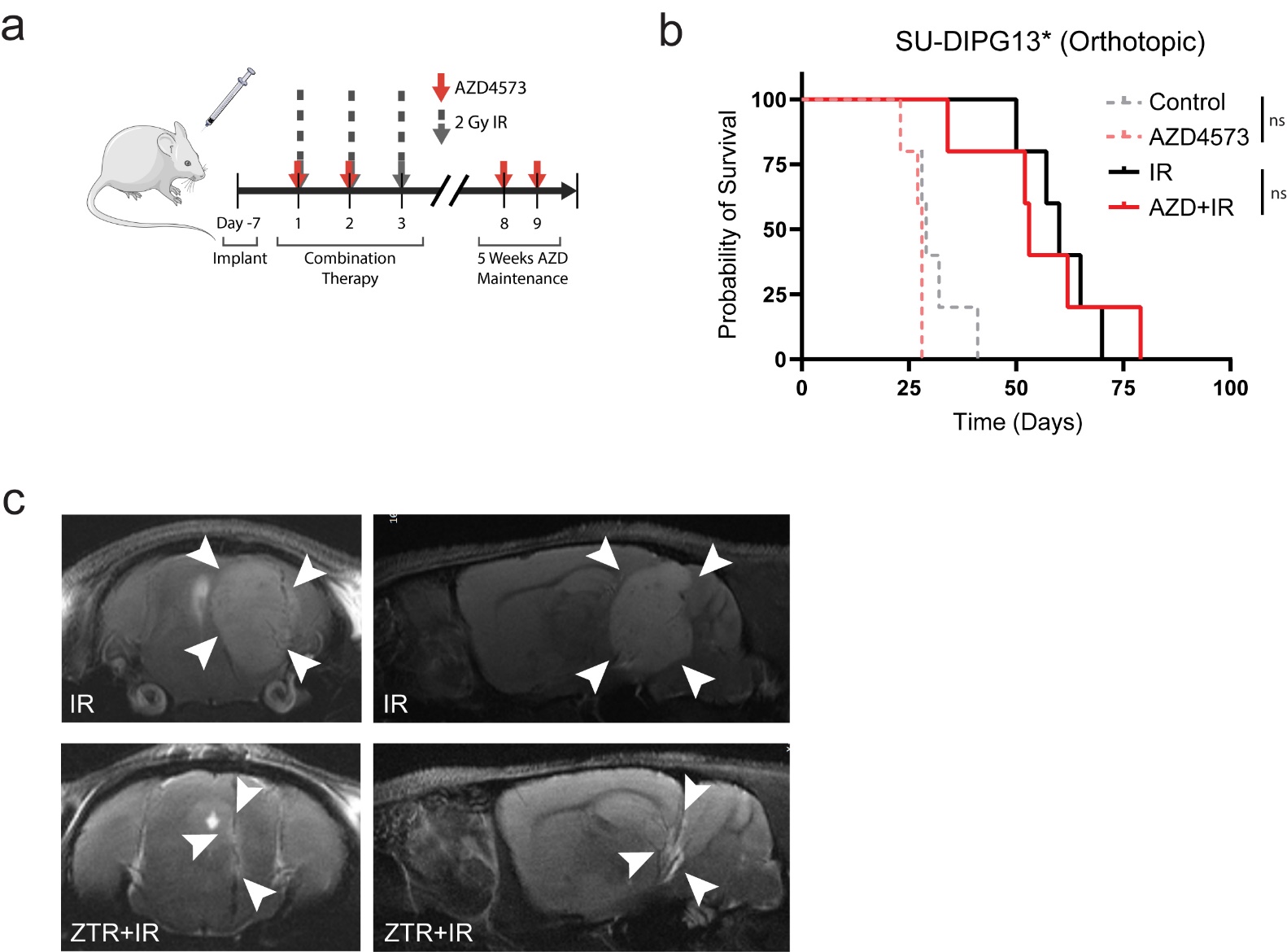


**Supplementary Figure 16.** AZD4573 is ineffective against intracranial model of DIPG. **a.** Schematic represents the treatment schedule of SU-DIPG13* xenografts with either AZD4573 (15/15 mg/kg biweekly administered intraperitoneally), radiotherapy (2 Gy x 3 fractions), or combination. **b.** Kaplan-Meier survival analysis of orthotopic xenograft cohorts from (a) receiving indicated treatments (control n=5, AZD4573 n=5, IR n=5, AZD+IR n=5). Source data are provided as a Source Data file.


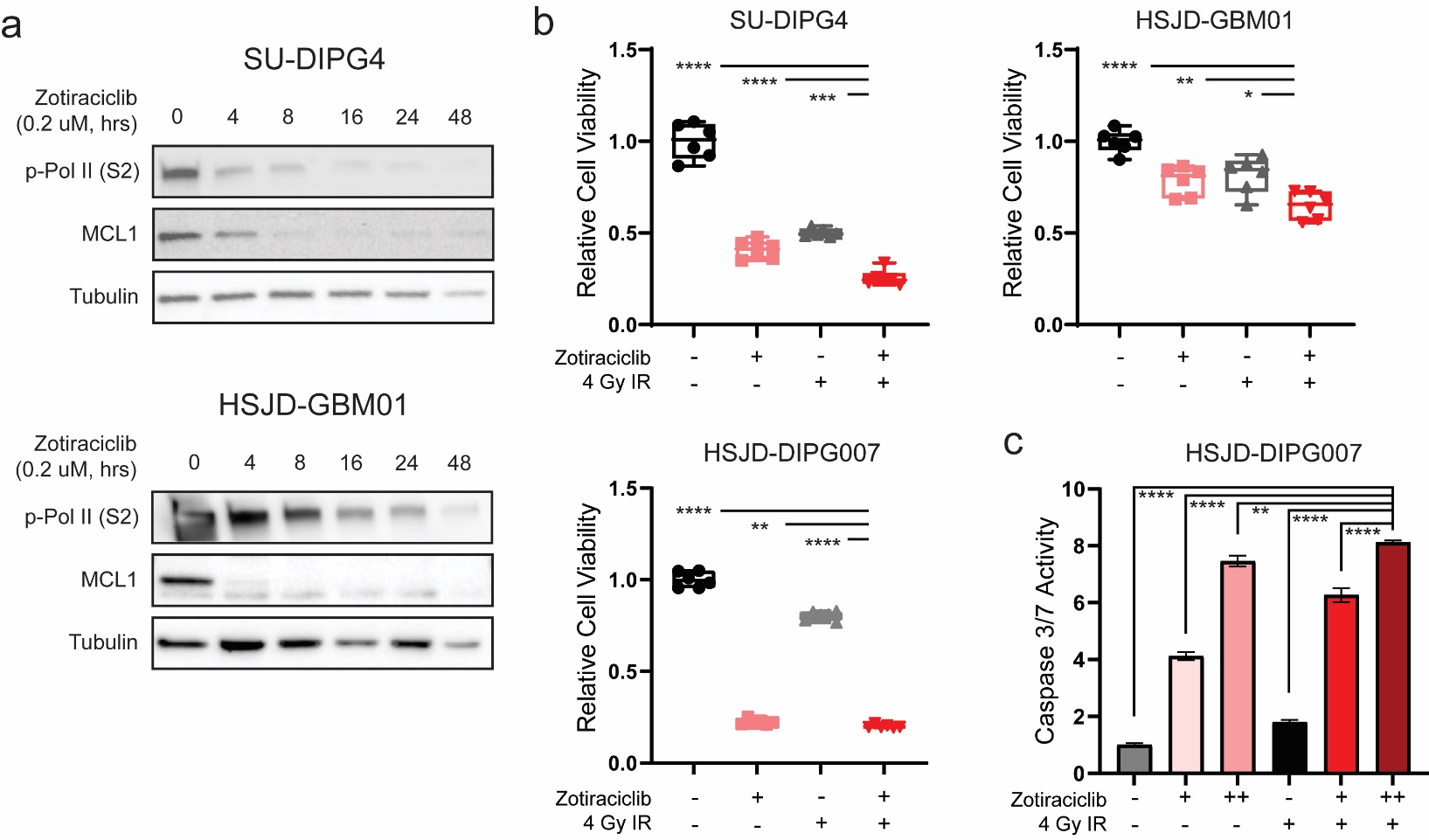


**Supplementary Figure 17.** CDK9 inhibitory activity of zotiraciclib. **a.** Western blot analysis of p-Pol II (Ser 2) and MCL1 after indicated exposure times to 0.2 µM zotiraciclib. Data represent single experiment. **b.** Cell viability measured at 3 days following 24-hour exposure to 0.2 µM zotiraciclib +/- 4 Gy IR. Box whiskers represent min to max range of replicates. Quantitative comparisons reflect two-tailed Student’s t-test (* p=<0.05, ** p=<0.01, *** p=<0.001, **** p=<0.0001), n=6 biologically independent replicates. **c.** Caspase 3/7 activity in HSJD-DIPG007 cells treated with 4 Gy IR, ZTR 80 nM (+) to 100 nM (++), or combination. Quantitative comparisons reflect two-tailed Student’s t-test (** p=<0.01, **** p=<0.0001), mean ±SEM of n=6 (control, IR) or n=12 (ZTR +) biologically independent replicates. Source data are provided as a Source Data file.


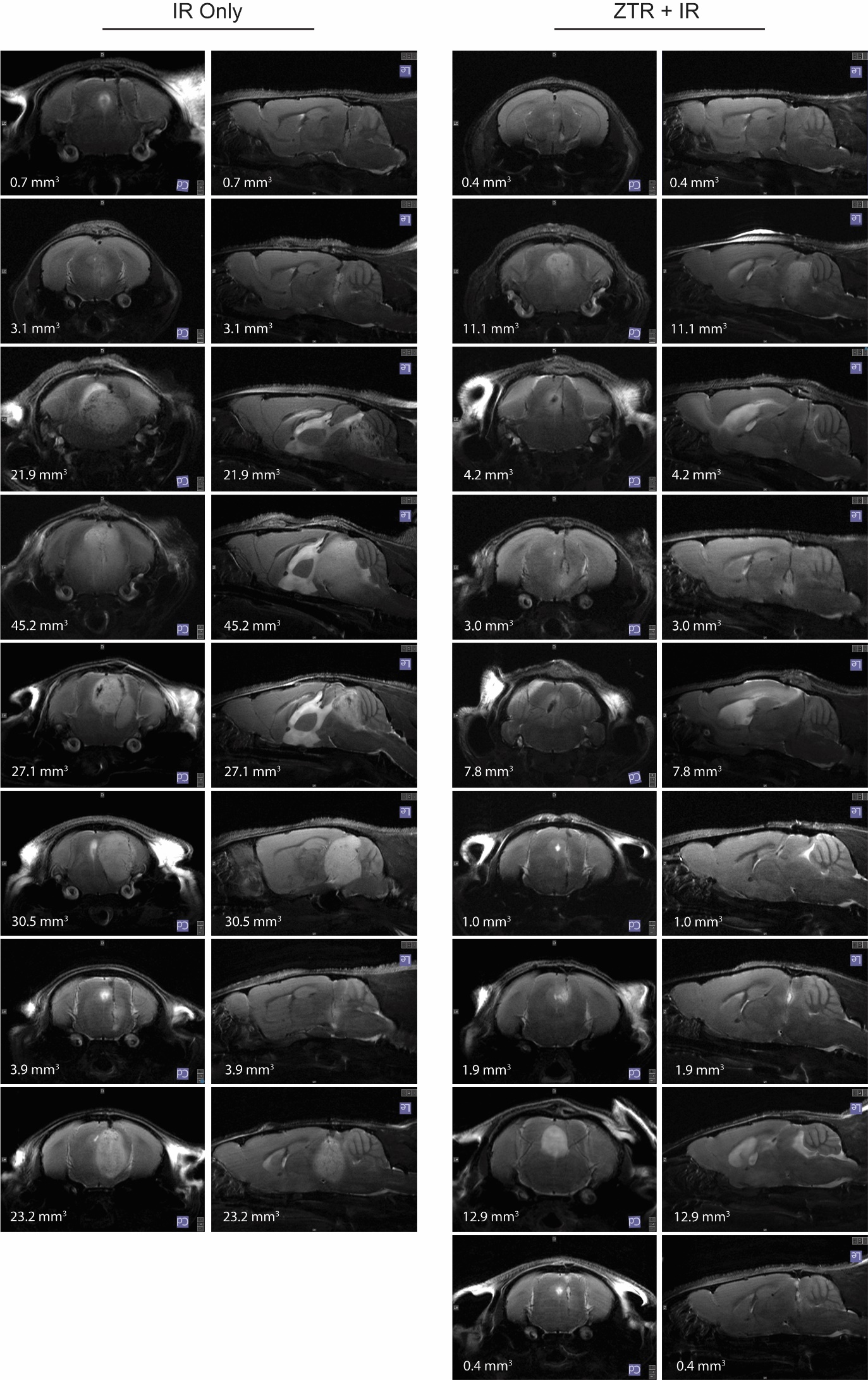


**Supplementary Figure 18.** Zotiraciclib exhibits radiographic anti-tumor effect. Representative axial (left) or sagittal (right) T2-weighted turboRARE axial MRI sequences of IR- or ZTR+IR-treated mice. Three-dimensional tumor volume in text insert.

**
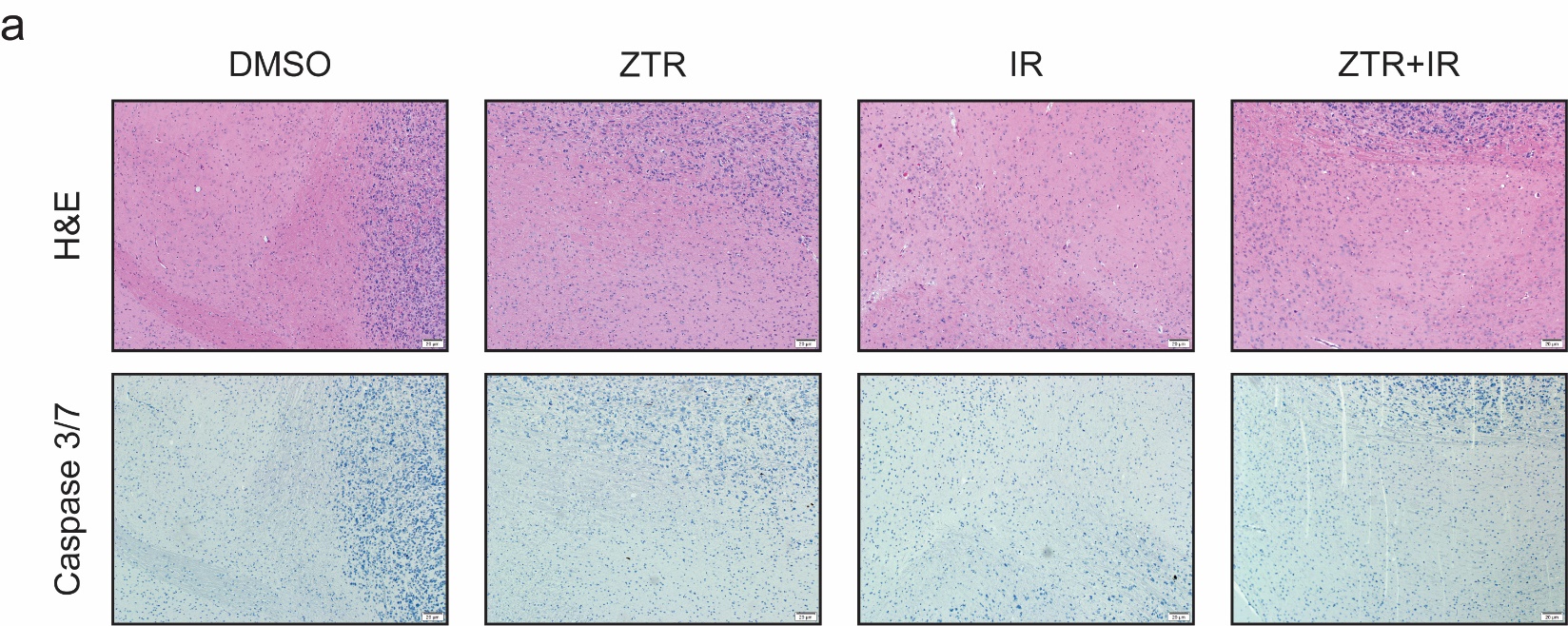
**

**Supplementary Figure 19.** Zotiraciclib therapy does not increase radiation toxicity to surrounding brain. **a.** H&E stains (top) and caspase 3/7 immunohistochemistry (bottom) from tumor margin of indicated mice demonstrates no evidence of histologic necrosis or induction of apoptosis in surrounding normal brain. Images are representative of n=3 biologically independent animals imaged 3 fields per replicate.
