# Supplementary material for: Rapid P-TEFb-dependent transcriptional reorganization underpins the glioma adaptive response to radiotherapy
Source: Nat Commun. 2024 May 30;15:4616. doi: 10.1038/s41467-024-48214-3 (PMC11139976; doi:10.1038/s41467-024-48214-3)
Supplement: Supplementary file 2 — Description of Additional Supplementary Files [file 41467_2024_48214_MOESM2_ESM.pdf]

Description of Additional Supplementary Files

Supplementary Data 1. Functional enrichment analysis of ATAC-seq. Enrichment defined by Metascape using hypergeometric test and Benjamini-Hochberg P value correction algorithm.

Supplementary Data 2. Functional enrichment analysis of H3K27ac. Enrichment defined by Metascape using hypergeometric test and Benjamini-Hochberg P value correction algorithm.

Supplementary Data 3. Functional enrichment analysis of RNA-seq. Enrichment defined by Metascape using hypergeometric test and Benjamini-Hochberg P value correction algorithm.

Supplementary Data 4. Functional enrichment analysis of CDK9-dependent gene expression. Enrichment defined by Metascape using hypergeometric test and Benjamini-Hochberg P value correction algorithm.

Supplementary Data 5. STR profiling of cell culture models.
