# Supplementary material for: Rapid P-TEFb-dependent transcriptional reorganization underpins the glioma adaptive response to radiotherapy
Source: Nat Commun. 2024 May 30;15:4616. doi: 10.1038/s41467-024-48214-3 (PMC11139976; doi:10.1038/s41467-024-48214-3)
Supplement: Supplementary file 8 — Reporting Summary [file 41467_2024_48214_MOESM8_ESM.pdf]

nature portfolio

Corresponding author(s): Nathan Dahl

Last updated by author(s): 4/4/2024

Reporting Summary

Nature Portfolio wishes to improve the reproducibility of the work that we publish. This form provides structure for consistency and transparency in reporting. For further information on Nature Portfolio policies, see our Editorial Policies and the Editorial Policy Checklist.

Statistics

For all statistical analyses, confirm that the following items are present in the figure legend, table legend, main text, or Methods section.

n/a	Confirmed	
☐	☒	The exact sample size (*n*) for each experimental group/condition, given as a discrete number and unit of measurement
☐	☒	A statement on whether measurements were taken from distinct samples or whether the same sample was measured repeatedly
☐	☒	The statistical test(s) used AND whether they are one- or two-sided<br>*Only common tests should be described solely by name; describe more complex techniques in the Methods section.*
☒	☐	A description of all covariates tested
☒	☐	A description of any assumptions or corrections, such as tests of normality and adjustment for multiple comparisons
☐	☒	A full description of the statistical parameters including central tendency (e.g. means) or other basic estimates (e.g. regression coefficient) AND variation (e.g. standard deviation) or associated estimates of uncertainty (e.g. confidence intervals)
☐	☒	For null hypothesis testing, the test statistic (e.g. *F*, *t*, *r*) with confidence intervals, effect sizes, degrees of freedom and *P* value noted<br>*Give P values as exact values whenever suitable.*
☒	☐	For Bayesian analysis, information on the choice of priors and Markov chain Monte Carlo settings
☒	☐	For hierarchical and complex designs, identification of the appropriate level for tests and full reporting of outcomes
☒	☐	Estimates of effect sizes (e.g. Cohen's *d*, Pearson's *r*), indicating how they were calculated

Our web collection on statistics for biologists contains articles on many of the points above.

Software and code

Policy information about availability of computer code

Data collection	Live cell imaging was collected using the Incucyte S3 system and analyzed on manufacturer's software package. Fixed immunofluorescSnce images we acquired on the Keyence Fluorescent System and analyzed in ImageJ.
Data analysis	Unless indicated otherwise in the figure legend, all in vitro data are presented as mean ± SEM. In vitro assays were performed with a minimum of three independent samples, and key experiments were successfully replicated with independent samples on separate days. For quantitative comparisons, significance was defined as n.s. p>0.05, * p=<0.05, ** p=<0.01, *** p=<0.001, **** p=<0.0001. All statistical analysis was performed on GraphPad Prism 9.0 software (GraphPad, La Jolla, CA). Kaplan-Meier survival curve comparisons were performed by log-rank (Mantel-Cox) test using GraphPad Prism 9.0 software.

For manuscripts utilizing custom algorithms or software that are central to the research but not yet described in published literature, software must be made available to editors and reviewers. We strongly encourage code deposition in a community repository (e.g. GitHub). See the Nature Portfolio guidelines for submitting code & software for further information.

Data

Policy information about availability of data

All manuscripts must include a data availability statement. This statement should provide the following information, where applicable:

- Accession codes, unique identifiers, or web links for publicly available datasets
- A description of any restrictions on data availability
- For clinical datasets or third party data, please ensure that the statement adheres to our policy

The accession number for the raw and processed data reported in this paper is GEO: GSE227797.

Human research participants

Policy information about studies involving human research participants and Sex and Gender in Research.

Reporting on sex and gender	N/A
Population characteristics	N/A
Recruitment	N/A
Ethics oversight	N/A

Note that full information on the approval of the study protocol must also be provided in the manuscript.

Field-specific reporting

Please select the one below that is the best fit for your research. If you are not sure, read the appropriate sections before making your selection.

[x] Life sciences [ ] Behavioural & social sciences [ ] Ecological, evolutionary & environmental sciences

For a reference copy of the document with all sections, see nature.com/documents/nr-reporting-summary-flat.pdf

Life sciences study design

All studies must disclose on these points even when the disclosure is negative.

Sample size	Sample size determined by prior experiments by our lab and others using the in vivo model system and anticipated effect size.
Data exclusions	No data was excluded from the analysis.
Replication	To ensure reproducibility and rigor, all in vitro experiments were conducted with minimum 3 independent biological replicates. Key experiments were repeated 2-3 independent times by different laboratory personnel, when possible.
Randomization	As described in methods, all animals were ordered by bioluminescent flux approximately 7 days post-injection and randomized sequentially into treatment groups.
Blinding	For animal experiments, treating personnel were not blinded to treatment administered. Animals were regularly assessed for endpoint criteria by veterinary technicians blinded to treatment group assignment. All MRI acquisitions and image analyses were performed by a radiologist blinded to the treatment assignment of the mice. For in vitro functional studies, blinding is not relevant given clearly distinguishable treatments and reporter effects.

Reporting for specific materials, systems and methods

We require information from authors about some types of materials, experimental systems and methods used in many studies. Here, indicate whether each material, system or method listed is relevant to your study. If you are not sure if a list item applies to your research, read the appropriate section before selecting a response.

Materials & experimental systems

n/a	Involved in the study
☐	☒ Antibodies
☐	☒ Eukaryotic cell lines
☒	☐ Palaeontology and archaeology
☐	☒ Animals and other organisms
☒	☐ Clinical data
☒	☐ Dual use research of concern

Methods

n/a	Involved in the study
☐	☒ ChIP-seq
☒	☐ Flow cytometry
☐	☒ MRI-based neuroimaging

Antibodies

Antibodies used	CDK9 (Abcam [EPR3119Y] (ab76320)) (1 to 1000)<br>CDK9 (Cell Signaling, [C12F7] #2316) (1 to 1000)<br>phospho-Rbp1 CTD (Ser2): Cell Signaling (E1Z3G) #13499 (1 to 1000)<br>phospho-Rbp1 CTD (Ser5): Cell Signaling (D9N5I) #13523S (1 to 1000)<br>Pol II (Rpb1 CTD): Cell Signaling, (4H8) #2629S (1 to 1000)<br>α-Tubulin: Cell Signaling (DM1A) (#3873S) (1 to 10,000)<br>Alexa Fluor 488 Mouse anti-H2AX: BD Pharmigen (Ps139) 560445 (1 to 10)<br>H3K27Ac: Abcam Cat. #ab4729 (1ug/ml for Western, 2ug for 25ug chromatin for ChIP-seq)<br>BRD4 (EpiCypher, 13-2003) (0.5ug per reaction for CUT&RUN)<br>ENL (Cell Signaling, [D9M4B] #14893) (0.5ug per reaction for CUT&RUN)<br>LARP7 (abcam # ab134746) (1 to 2000)<br>MEPCE (abcam #185991) (1 to 2000)<br>HEXIM1 (Everest Biotech, #EB06964) (1 to 1000)<br>anti-rabbit light chain (Jackson ImmunoResearch, 211-002-171) (1 to 2000)<br>p-BRG1 (Cell Signaling [Ser1627/1631] [E2N9V] #58034) (1 to 1000)<br>BRG1 (Cell Signaling [D1Q7F] #49360) (1 to 1000)
Validation	All antibodies utilized in this study have been previously validated by the manufacturers for their respective use. Manufacturer information including clone and catalog number are included in the Materials and Methods sections. CDK9 antibodies were additionally validated in human knockdown cells with qPCR correlation.

Eukaryotic cell lines

Policy information about cell lines and Sex and Gender in Research

Cell line source(s)	No commercially available cell lines were utilized for this research. Eukaryotic lines were developed and shared from the following sources:<br>SU-DIPG4 and SU-DIPG13 were provided by Michelle Monje (Stanford University)<br>HSJD-DIPG007, HSJD-GBM001, and HSJD-GBM002 were provided by Angel Montero Carcaboso (Sant Joan de Déu)<br>SU-pcGBM2 was provided by Siddhartha Mitra (Stanford University, University of Colorado)<br>SF8268 and SF7761 were provided by Nalin Gupta (University of California, San Francisco)
Authentication	Patient derived cell lines were routinely monitored by STR analysis and compared to original values upon receipt at our institution. STR profiling is submitted as supplementary table 3.
Mycoplasma contamination	All cells are routinely tested for mycoplasma contamination, with no contamination detected.
Commonly misidentified lines<br>(See ICLAC register)	No commonly misidentified cell lines were used for this study.

Animals and other research organisms

Policy information about studies involving animals; ARRIVE guidelines recommended for reporting animal research, and Sex and Gender in Research

Laboratory animals	Strains: NOD SCID gamma mice were used for this study
Wild animals	No wild animals used for this study
Reporting on sex	All animal studies were conducted with a mixture of male and female mice. Mice were randomized to treatment groups to ensure an equal distribution according to sex. No differences in outcomes were observed on the basis of sex.
Field-collected samples	No field-collected samples were used for this study.
Ethics oversight	University of Colorado Institutional Animal Care and Use Committee (IACUC) approval was obtained and maintained throughout the

conduct of the study.

Note that full information on the approval of the study protocol must also be provided in the manuscript.

ChIP-seq

Data deposition

- [x] Confirm that both raw and final processed data have been deposited in a public database such as GEO.
- [x] Confirm that you have deposited or provided access to graph files (e.g. BED files) for the called peaks.

Data access links<br>*May remain private before publication.*	Data for this manuscript is available at: GSE227797. As this accession is private pending publication, reviewers may use the following token: yzmxygowhfmjvqr
Files in database submission	The raw and processed data for the following files are available in the above GEO:<br>SU-DIPG4: H3K27ac ChIP-seq - Control Replicate 1<br>SU-DIPG4: H3K27ac ChIP-seq - Control Replicate 2<br>SU-DIPG4: H3K27ac ChIP-seq - Control Input 1<br>SU-DIPG4: H3K27ac ChIP-seq - Control Input 2<br>SU-DIPG4: H3K27ac ChIP-seq - IR Replicate 1<br>SU-DIPG4: H3K27ac ChIP-seq - IR Replicate 2<br>SU-DIPG4: H3K27ac ChIP-seq - IR Input 1<br>SU-DIPG4: H3K27ac ChIP-seq - IR Input 2<br>SU-DIPG4: H3K27ac ChIP-seq - IR+AZD Replicate 1<br>SU-DIPG4: H3K27ac ChIP-seq - IR+AZD Replicate 2<br>SU-DIPG4: H3K27ac ChIP-seq - IR+AZD Input 1<br>SU-DIPG4: H3K27ac ChIP-seq - IR+AZD Input 2<br><br>SU-DIPG4: ATAC-seq - Control Replicate 1<br>SU-DIPG4: ATAC-seq - Control Replicate 2<br>SU-DIPG4: ATAC-seq - IR Replicate 1<br>SU-DIPG4: ATAC-seq - IR Replicate 2<br>SU-DIPG4: ATAC-seq - IR+AZD Replicate 1<br>SU-DIPG4: ATAC-seq - IR+AZD Replicate 2<br><br>SU-DIPG4: p-Pol II CUT&RUN - Control Replicate 1<br>SU-DIPG4: p-Pol II CUT&RUN - Control Replicate 2<br>SU-DIPG4: p-Pol II CUT&RUN - Control Replicate 3<br>SU-DIPG4: IgG CUT&RUN - Control Replicate 1<br>SU-DIPG4: IgG CUT&RUN - Control Replicate 2<br>SU-DIPG4: p-Pol II CUT&RUN - IR Replicate 1<br>SU-DIPG4: p-Pol II CUT&RUN - IR Replicate 2<br>SU-DIPG4: p-Pol II CUT&RUN - IR Replicate 3<br>SU-DIPG4: IgG CUT&RUN - IR Replicate 1<br>SU-DIPG4: IgG CUT&RUN - IR Replicate 2<br>SU-DIPG4: p-Pol II CUT&RUN - IR+AZD Replicate 1<br>SU-DIPG4: p-Pol II CUT&RUN - IR+AZD Replicate 2<br>SU-DIPG4: p-Pol II CUT&RUN - IR+AZD Replicate 3<br>SU-DIPG4: IgG CUT&RUN - IR+AZD Replicate 1<br>SU-DIPG4: IgG CUT&RUN - IR+AZD Replicate 2<br><br>SU-DIPG4: RNA-seq - Control Replicate 1<br>SU-DIPG4: RNA-seq - Control Replicate 2<br>SU-DIPG4: RNA-seq - Control Replicate 3<br>SU-DIPG4: RNA-seq - IR Replicate 1<br>SU-DIPG4: RNA-seq - IR Replicate 2<br>SU-DIPG4: RNA-seq - IR Replicate 3<br>SU-DIPG4: RNA-seq - IR+AZD Replicate 1<br>SU-DIPG4: RNA-seq - IR+AZD Replicate 2<br>SU-DIPG4: RNA-seq - IR+AZD Replicate 3<br><br>SF8628: RNA-seq - Control Replicate 1<br>SF8628: RNA-seq - Control Replicate 2<br>SF8628: RNA-seq - Control Replicate 3<br>SF8628: RNA-seq - IR Replicate 1<br>SF8628: RNA-seq - IR Replicate 2<br>SF8628: RNA-seq - IR Replicate 3<br>SF8628: RNA-seq - IR+AZD Replicate 1<br>SF8628: RNA-seq - IR+AZD Replicate 2

SF8628: RNA-seq - IR+AZD Replicate 3

HSJD-DIPG007: RNA-seq - Control Replicate 1
HSJD-DIPG007: RNA-seq - Control Replicate 2
HSJD-DIPG007: RNA-seq - Control Replicate 3
HSJD-DIPG007: RNA-seq - IR Replicate 1
HSJD-DIPG007: RNA-seq - IR Replicate 2
HSJD-DIPG007: RNA-seq - IR Replicate 3
HSJD-DIPG007: RNA-seq - IR+AZD Replicate 1
HSJD-DIPG007: RNA-seq - IR+AZD Replicate 2
HSJD-DIPG007: RNA-seq - IR+AZD Replicate 3

Genome browser session (e.g. UCSC)

NA

Methodology

Replicates

All ChIP-seq and ATAC-seq experiments were performed in independent biological duplicates. All CUT&RUN and RNA-seq experiments were performed in independent biological triplicate.

Sequencing depth

Flow cells were loaded to obtain 30-40 million paired end 150bp reads per sample.

Antibodies

H3K27Ac: Abcam Cat. #ab4729
phospho-Rbp1 CTD (Ser2): Cell Signaling (E1Z3G) #13499
BRD4 (EpiCypher, 13-2003)
ENL (Cell Signaling, [D9M4B] #14893)

Peak calling parameters

Peaks were called using MACS2 (v2.1.2) using ENCODE recommendations. IDR was used to identify the reproducible peaks between the replicates. Further processing of the peak data was performed in R, using in particular the following tools: valR and DiffBind.

Data quality

The quality of the fastq files was accessed using FastQC and MultiQC. Illumina adapters and low-quality reads were filtered out using BBDuk (http://jgi.doe.gov/data-and-tools/bb-tools). Bowtie2 (v.2.3.4.3) was used to align the sequencing reads to the hg38 reference human genome. Samtools (v.1.11) was used to select the mapped reads (samtools view -b - q 30) and sort the bam files. PCR duplicates were removed using Picard MarkDuplicates tool (http://broadinstitute.github.io/picard/). The normalization ratio for each sample was calculated by dividing the number of uniquely mapped human reads of the sample with the lowest number of reads by the number of uniquely mapped human reads of each sample. These normalization ratios were used to randomly sub-sample reads to obtain the same number of reads for each sample using using samtools view -s.

Software

FastQC, MultiQC, BBDuk, Bowtie2, Samtools, Picard MarkDuplicates, MACS2, IDR, valR, DiffBind, Bedtools genomecov, deepTools bamCoverage, ngs.plot

Magnetic resonance imaging

Experimental design

Design type

Xenograft tumor monitoring

Design specifications

NA

Behavioral performance measures

NA

Acquisition

Imaging type(s)

Structural

Field strength

9.4 Tesla

Sequence & imaging parameters

Non-gadolinium multi-sequential MRI protocol was applied to acquire (i) high-resolution 3D T2-weighted turboRARE; (ii) sagittal FLAIR;

Area of acquisition

Whole brain

Diffusion MRI ☐ Used ☒ Not used

Preprocessing

Preprocessing software

All MRI acquisitions and image analysis were performed using Bruker ParaVision 360NEO software.

Normalization

NA

Normalization template	NA
Noise and artifact removal	NA
Volume censoring	NA

Statistical modeling & inference

Model type and settings	NA
Effect(s) tested	NA

Specify type of analysis: ☒ Whole brain ☐ ROI-based ☐ Both

Statistic type for inference (See Eklund et al. 2016)	NA
Correction	NA

Models & analysis

n/a	Involved in the study
☒	☐ Functional and/or effective connectivity
☒	☐ Graph analysis
☒	☐ Multivariate modeling or predictive analysis
